# Discovery of novel natural products as dual MNK/PIM inhibitors for acute myeloid leukemia treatment: Pharmacophore modeling, molecular docking, and molecular dynamics studies

**DOI:** 10.3389/fchem.2022.975191

**Published:** 2022-07-22

**Authors:** Linda M. Mohamed, Maha M. Eltigani, Marwa H. Abdallah, Hiba Ghaboosh, Yousef A. Bin Jardan, Osman Yusuf, Tilal Elsaman, Magdi A. Mohamed, Abdulrahim A. Alzain

**Affiliations:** ^1^ Department of Pharmaceutical Chemistry, Faculty of Pharmacy, University of Gezira, Gezira, Sudan; ^2^ Department of Pharmaceutics, Faculty of Pharmacy, University of Gezira, Gezira, Sudan; ^3^ Department of Pharmaceutics, College of Pharmacy, King Saud University, Riyadh, Saudi Arabia; ^4^ Department of Pharmaceutical Chemistry, College of Pharmacy, Jouf University, Sakaka, Saudi Arabia

**Keywords:** acute myeloid leukemia, natural products, MNK-2, Pim-2, molecular docking, molecular dynamics

## Abstract

MNK-2 and PIM-2 kinases play an indispensable role in cell proliferation signaling pathways linked to tyrosine kinase inhibitors resistance. In this study, pharmacophore modeling studies have been conducted on the co-crystalized ligands of MNK-2 and PIM-2 enzyme crystal structures to determine the essential features required for the identification of potential dual inhibitors. The obtained pharmacophore features were then screened against a library of 270,540 natural products from the ZINC database. The matched natural molecules were docked into the binding sites of MNK-2 and PIM-2 enzymes. The compounds with high docking scores with the two enzymes were further subjected to MM-GBSA calculations and ADME prediction. This led to the identification of compound 1 (ZINC000085569211), compound 2 (ZINC000085569178), and compound 3 (ZINC000085569190), with better docking scores compared to the reference co-crystallized ligands of MNK-2 and PIM-2. Moreover, compounds 1‒3 displayed better MM-GBSA binding free energies compared to the reference ligands. Finally, molecular dynamics (MD) study was used to assess the interaction stability of the compounds with MNK-2. To this end, compounds 1 and 3 bound strongly to the target during the whole period of MD simulation. The findings of the current study may further help the researchers in the discovery of novel molecules against MNK-2 and PIM-2.

## 1 Introduction

Acute myeloid leukemia (AML) is a highly aggressive hematological cancer characterized by a variety of mutations and cytogenetic abnormalities that lead to a large number of immature hematopoietic cells that proliferate and accumulate in the blood (Yen et al., 2022). It is very common among adults and young people whose rate increases with age (Key Statistics for Acute Myeloid, 2022). Currently, AML patients are treated with a standard (3 + 7) treatment regimen that includes daunorubicin and citarabine, both of which have been linked to serious side effects, yet relapse remains a concern for these patients (Kantarjian et al., 2021). Although this is resolved through stem cell transplantation, it is linked to increased treatment-related morbidity and mortality (high-grade hematological toxicities are common), especially in elderly patients (Martelli et al., 2007; Gao et al., 2018; Zhang et al., 2020). Consequently, a unique and successful AML treatment strategy is needed. In this context, targeted treatment and kinase inhibition, such as provirus integration in Moloney murine leukemia viral kinases, have replaced chemotherapy in AML treatment (PIMs) (Kapelko-slowik et al., 2016; Rebello et al., 2017).

PIM kinases are proto-oncogenic serine/threonine kinases that comprise three PIM 1, 2, and 3 homologous proteins with high levels of functional similarity, however, they differ in their distribution in tissues (Jiménez-garcía et al., 2016; Han et al., 2021). Both isoforms 1 and 2 are upregulated in several malignant hematological tumors, including AML, chronic lymphocytic leukemia, acute lymphoblastic leukemia, multiple myeloma, and non-Hodgkin lymphoma (Keeton et al., 2014). They promote AML cell survival by phosphorylating the Bcl-2 cell death antagonist (BAD) repealing its inhibitory binding to the anti-apoptotic Bcl-xL protein (Wang et al., 2019; Maney et al., 2021). PIMs share several substrates with the AKT pathway, including PRAS40, which inhibits mTORC1 and, as a result, protein translation by the mTORC1-substrate (Kapelko-slowik et al., 2016; Rebello et al., 2017). PIM-2 has been identified as a key regulator of 4EBP1 and cap-dependent translation in AML, with the ability to maintain translation in the presence of a mTORC1 inhibitor, according to Keaton *et al* (Keeton et al., 2014; Zhang et al., 2018). Thus, inhibiting PIM kinase activity opens up a new therapeutic avenue for the treatment of AML (Keeton et al., 2014; Zhang et al., 2018). Unfortunately, the majority of PIM inhibitors show little or no effect when administered as a single drug, due to the rapid resistance produced by redundancy or feedback signaling (Nawijn et al., 2011; Tursynbay et al., 2016). Therefore, it is urgent to identify drug combinations to prevent resistance to therapy such as inhibitors of PIM kinase in combination with inhibitors of MNK (Han et al., 2021).

MNK1 and MNK2 (MAPK-interacting kinases 1 and 2) are the only kinases that phosphorylate the eukaryotic translation initiation factor 4E (eIF4E), which is required for cell development, death, and metastasis (Dreas et al., 2017; Han et al., 2021). Such a phosphorylation is the rate-limiting factor in the initiation of mRNA translation and is important in the malignant transformation process (Fischer, 2009). It is worth noting that eIF4E’s oncogenic activity is important in the RAS/RAF/ERK and PI3K/AKT/mTOR pathways, both of which are active in AML (Wang et al., 2019). In addition, eIF4E is strongly expressed in AML cells; in particular in the subtype M4/M5 of AML (Assouline et al., 2016). Due to the participation of eIF4E in both the RAS/RAF/ERK and the PI3K/AKT/mTOR pathways, inhibiting its phosphorylation could decrease imipenemase (IMP) linked resistance. Therefore, PIM inhibition in combination with MNKs inhibitors may have a synergistic effect on AML cells (Han et al., 2021). Inhibition of both MNK and PIM is less likely to be associated with adverse side effects, and they may have minimal or no hematopoietic defects in mice (Kapelko-slowik et al., 2016).

Natural products (NPs) have shown great activity against various diseases; their use is explained by many advantages, including antiapoptotic, anti-inflammatory, cardioprotective, cosmological use, and others (Scotti et al., 2017; Koch et al., 2019; Antiviral, 2020; Tao et al., 2020; Wu et al., 2021). In recent years, NPs have been important sources of anticancer agents; not only for their structural variety and high activity but also for their greater selectivity and low side effects when compared to conventional cancer therapy (synthetic ones) (Khalid et al., 2016; Ruiz-Torres et al., 2018; Khalifa et al., 2019; Alam et al., 2021). Some NPs were also found to be effective in the treatment of MDR resistance (Banik et al., 2021). Camptothecin, paclitaxel, calyx, and thymoquinone are good examples of natural anticancer derivatives (Zhang et al., 2013; Zou et al., 2018; Mahmoud and Abdelrazek, 2019; Xu et al., 2020).

The *in silico* technique is one of the fundamental drug development methods that saves time and reduces cost (Bhadoriya et al., 2014; Idris et al., 2020; Alzain and Elbadwi, 2021; Elbadwi et al., 2021). It plays an essential role in the identification and screening of new or approved inhibitors with the prediction of their pharmacokinetic properties (KB et al., 2020).

In this study, we aimed to identify novel dual phytochemicals from the ZINC database against PIM-2 and MNK-2 using various *in silico* techniques such as multiple ligand-based pharmacophore approach, molecular docking, MM-GBSA calculations, ADME prediction, and MD simulations.

## 2 Methods

Schrödinger’s Maestro v 12.8 was used for computational investigations. MD simulations were performed using academic Desmond v.5 by D. E. Shaw Research.

### 2.1 Protein preparation

The three-dimensional (3D) structures of MNK-2 (ID: 2hw7) and PIM-2 (ID: 4x7q) with their co-crystallized ligands Staurosporine and 3YR, respectively, were obtained from the Protein Data Bank (Jauch et al., 2006; RCSB, 2010; Ishchenko et al., 2015). MNK-2 and PIM-2 were prepared by the protein preparation wizard tool of Maestro that refines and optimizes the protein structures (Madhavi Sastry et al., 2013). Missing residues and loops were corrected, hydrogen atoms were added, unwanted water molecules were removed, and seleno-methionine was converted to methionine in proper ionization status at 7.4 pH. Furthermore, MNK-2 and PIM-2 were energy minimized using the OPLS-3e force field.

### 2.2 Grid generation and ligand preparation

The position and size of the binding pocket were defined around the bound ligand structures of 2hw7 and 4x7q using the Glide Receptor Grid Generation tool of Maestro (Receptor Grid Generation Panel, 2014). This step facilitated the upcoming docking step by determining the site where the binding interaction took place. A library of 270540 natural compounds from the ZINC database was downloaded. The library was minimized to low-energy 3D structures using the MacroModel tool of Maestro (Muhamed and Vadstrup, 2014). The OPLS3e force field was used to minimize ligand energy using PRCG (Polak-Ribier Conjugate Gradient) with 2500 iterations.

### 2.3 E-pharmacophore model generation and screening

The two ligands from 2hw7 and 4x7q were extracted and they were used for generating a pharmacophore model using the Phase’s option “create pharmacophore model from multiple ligands” taking into account finding the best alignment and common features coinciding with at least 50% of fundamental ligands, to obtain pharmacophore features for these two ligands (Dixon et al., 2006).

The minimized ZINC database library (270,540 compounds) was screened by the Phase tool, throughout the five pharmacophore models to filter the molecules that match the full chemical features of the hypotheses.

### 2.4 Molecular docking

Bound ligands interacting with MNK-2 and PIM-2 structures were used to develop the pharmacophore hypothesis. Phase screening results were introduced into the Glide docking tool for the docking study, which was conducted to assess the affinity of binding of these compounds to receptors (Lyne et al., 2006). In other words, the ligands were filtered depending on their binding strength to the receptor by evaluating the docking at three levels; high virtual screening throughput (HTVS) fast and random screening stage, standard precision (SP), and extra precision (XP) that was most accurate. The compounds from the Phase screens were subjected to two docking steps. Initially, these compounds were subjected to HTVS and SP; among the hundred ligands, the best conformations of the SP results were again filtered by XP against MNK-2 and PIM-2. The two co-crystallized ligands were re-docked against their primary proteins to validate the docking procedure. The XP docking score in Glide was calculated using the following equation:

Docking score (Glide score) = a*vdW + b*Coul + Hbond +Lipo + Metal + RotB + site

To assess the validity of our docking model, the root mean square deviation (RMSD) values were calculated for the binding patterns of the reference ligands in crystal structure and after molecular docking with the two prepared proteins.

### 2.5 MM-GBSA calculation

The free binding energy of the top hits complex with MNK-2 and PIM-2 was further analyzed by Molecular Mechanics-Generalized Born and surface area (MM-GBSA) by the Prime tool of Maestro, which takes into account the influence of the solvent in the binding energy calculation (Vijayakumar et al., 2014). The energy calculation of the minimized structures was carried out on the VSGB solvation model and OPLS3e force field using the following equation:

ΔG(bind) = G_complex_–G_protein_–G_ligand_

Where G_complex_ is the energy of the protein-ligand complex, G_protein_ is the energy of the protein, and G_ligand_ is the energy of the ligand.

### 2.6 ADME prediction

Pharmacokinetic properties prediction for the two co-crystallized ligands and top hits were performed using the QikProp tool (QikProp Schrödinger, 2019). This method eliminates any compound that was predicted to have a high failure rate in further clinical studies based on filtered ligand kinetics characteristics. Various physicochemical and pharmacokinetic characteristics were calculated; QPlogBB indicates the permeability of the molecule to the brain, QPlogPo/w indicates the lipophilicity profile of molecules QPlogS predicts possible solubility, QPPCaco predicts the permeability of the molecule to cells, and human oral absorption, Lipinski’s rule of five, which is considered a crucial measure of drug-likeness. ADME of the highest XP docking hits was compared with co-crystalized ligands.

### 2.7 MD simulation

The best three hits in terms of XP docking scores and MM-GBSA binding free energy were submitted further for MD analysis using the Desmond software against MNK-2 (Suryanarayanan and Singh, 2015). Here, we examined the binding stability of ligand-protein complexes and identified the most potential interactions that took place. The MNK-2 system was processed for MD simulations. First, MNK-2 in complex with compounds was prepared for MD simulations using Desmond’s System Builder (Kumar et al., 2019). In which, the MNK-2 system was solvated in 17191 TIP3P water molecules in an orthorhombic box (10 Å × 10 Å × 10 Å). Salt was added in a specific concentration of Na^+^ and Cl^−^ charge, as; 59.228 mM (Total charge + 56) for Na^+^, while 50.767 mM (Total charge − 48) for Cl^−^. The system was then energy minimized and pre-equilibrated with the default settings. The MD production final stage was set for 100 ns with NPT at 300K by Nose-Hoover thermostat and 1.013 bar by Martyna-Tobias-Klein barostat. During stimulation, 2009 frames were obtained for each system. The MD trajectories for the system were analyzed with Maestro’s Simulation Interaction Diagram.

## 3 Results and discussion

**FIGURE 1:**
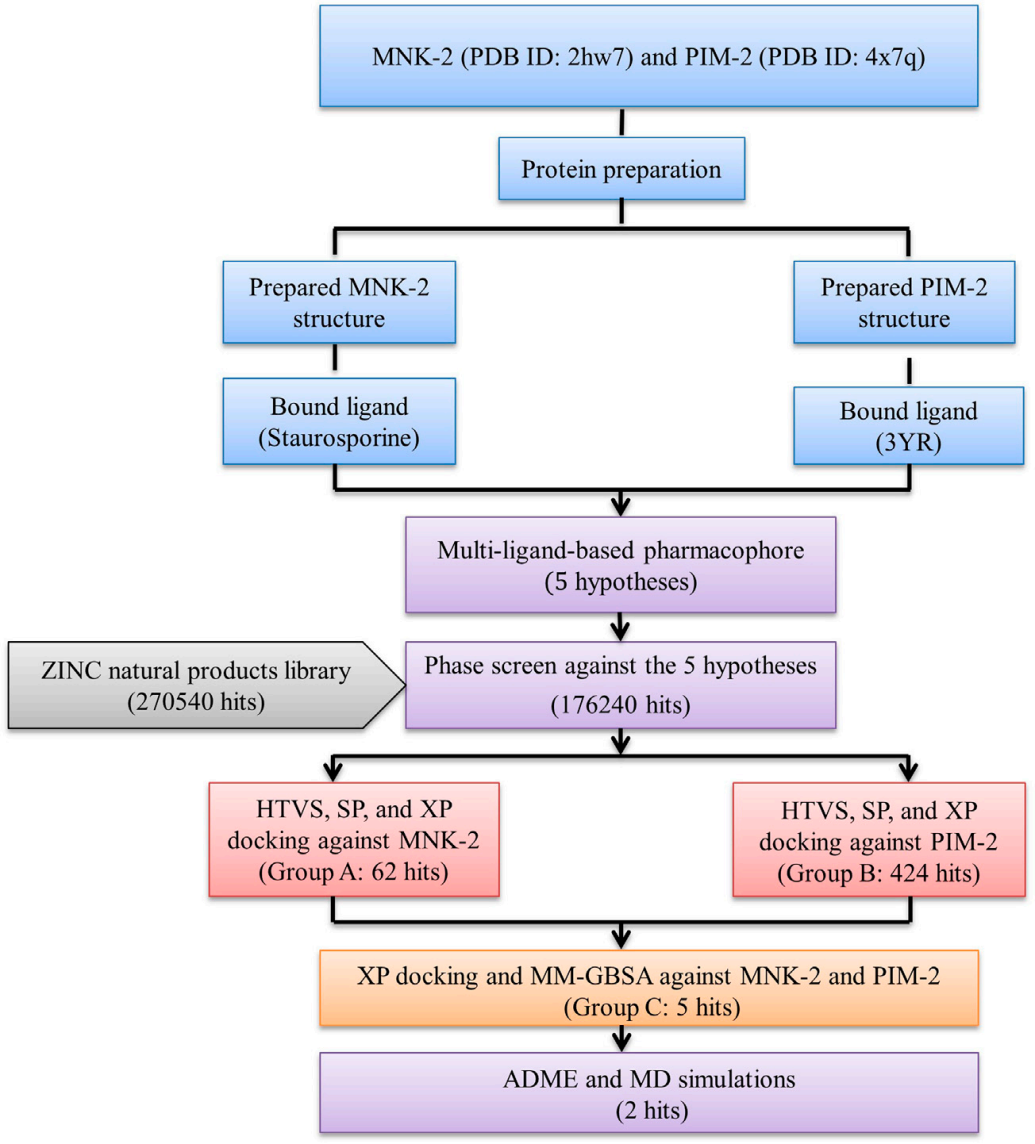
The overall work of the study.

The results were summarized as shown in Figure 1.

### 3.1 E-pharmacophore modeling and screening

Inhibition of both PIM and MNK kinases can have synergistic effects on AML cells (Han et al., 2021). Our study aimed to discover natural compounds to target two enzymes, MNK-2 and PIM-2, using a pharmacophore modeling strategy. Thus, we planned to generate a pharmacophore using naturally occurring ligands for both enzymes. To our knowledge, there is only one available MNK-2 crystal structure in which the co-crystallized ligand is a natural compound (PDB ID: 2hw7) which is Staurosporine. On the other hand, the literature revealed only two crystal structures for PIM-2 (PDB ID: 2IWI and 4X7Q), but none of them have a natural co-crystallized ligand. In this study, we selected 2hw7 for MNK-2 and 4x7q for PIM-2, as 2IWI is bound with ruthenium metal ligand (Ru1), which will complicate pharmacophore generation, library screening, and docking studies. Although it is a universal kinase and cannot be considered as an MNK-2 or PIM-2 specific inhibitor, we decided to start the study with the natural ligand Staurosporine. Findings of early docking and MM-GBSA calculations of this ligand with both proteins (MNK-2 and PIM-2) showed that the ligand has a very low binding affinity to PIM-2, as evidenced by both the docking score and the free binding energy (Table 1), suggesting that the non-specificity of Staurosporine may not be a major drawback of the study. Natural ligands’ screening was performed using the Zinc database which is freely available and contains a curated collection of natural compounds that excludes metabolites. Supplementary Figure S1 (supplementary file) displayed a representative of natural compounds used in this study.

**TABLE 1:** Docking scores and MM-GBSA values for the top 5 compounds with the two targets MNK-2 and PIM-2.

Compounds	MNK-2 (PDB ID: 2hw7)		PIM-2 (PDB ID: 4x7q)	
	XP GScore Kcal/mol (kcal/mol)	MMGBSA dG bind (kcal/mol)	XP GScore (kcal/mol)	MMGBSA dG bind (kcal/mol)
ZINC000085569211	−12.578	−61.55	−10.612	−47.5
ZINC000085569178	−12.174	−62.65	−10.907	−47.18
ZINC000085569190	−12.113	−64.73	−10.022	−47.72
ZINC000008879593	−11.596	−49.29	−10.312	−43.23
ZINC000012886855	−11.141	−50.35	−9.568	−43.19
Reference (2hw7)	−10.755	−44.22	−1.903	−2.42
Reference (4x7q)	−7.014	−55.4	−7.793	−70.37

**FIGURE 2:**
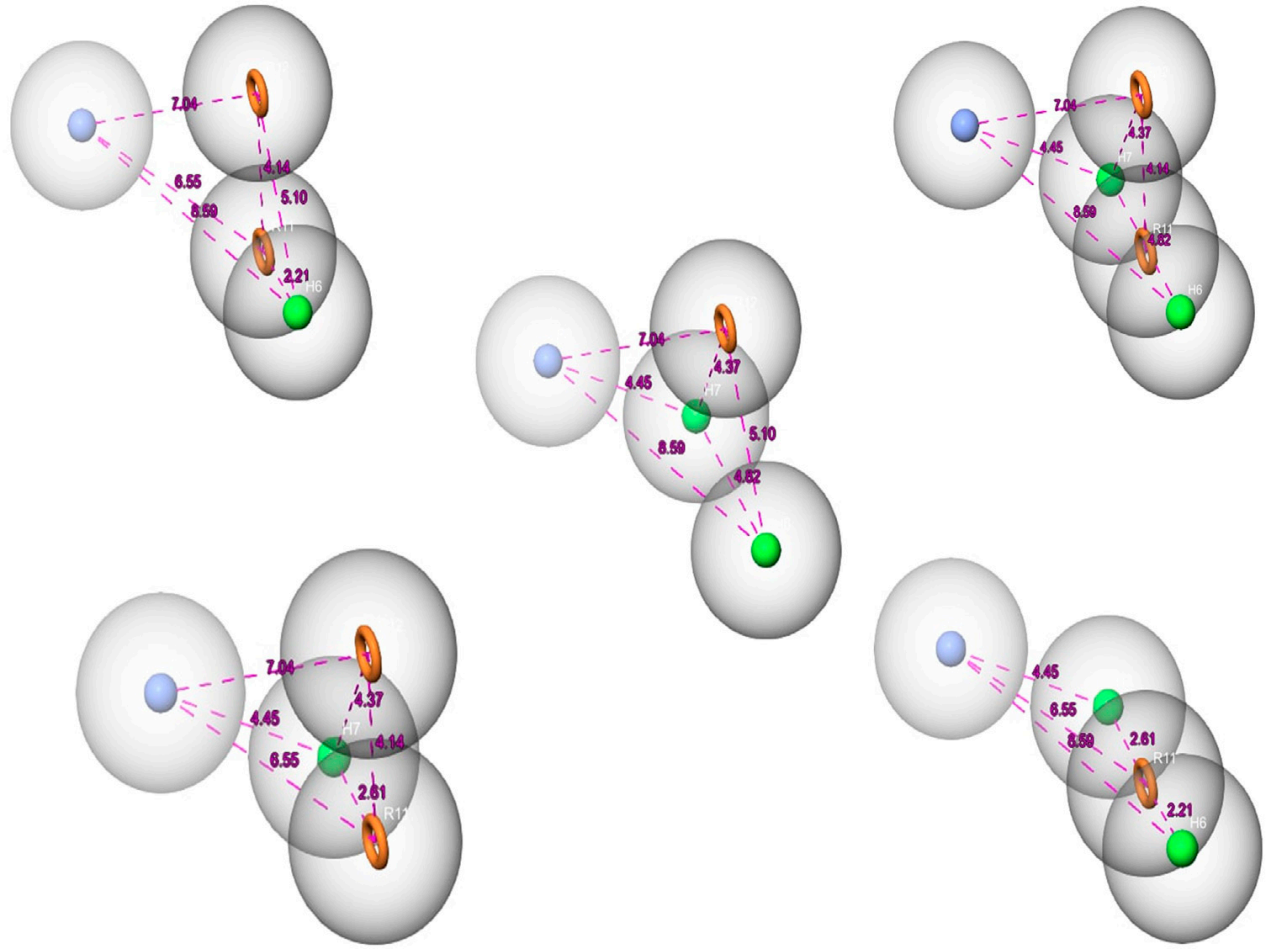
The pharmacophore hypothesis developed using the bound ligands with MNK-2 (ID: 2hw7) and PIM-2 (ID: 4x7q). The hypothesis was generated using “Generate hypothesis from multiple ligands” option of Phase software. All the distances are in Å unit. Pink sphere with arrow, hydrogen-bond acceptor (A); yellow open circle, aromatic ring (R); blue sphere with arrow, hydrogen-bond donor (D).

Over the last few years, the ligand pharmacophore model (LBPM) has been considered one of the most promising *in silico* techniques (Janežič et al., 2021; Lin et al., 2021). In this study, LBPM was constructed from the two co-crystallized ligands to create hybrid compounds having common properties for both ligands. Five pharmacophore hypotheses were studied and each hypothesis was represented by five properties, including the chemical characteristic of hydrophobic atoms (H), aromatic rings (R), and positive ionic (P), as shown in Figure 2.

The 270,540 natural compounds prepared from the ZINC database were screened against the five hypotheses. The compounds were set to match the full chemical features of these hypotheses. The total of Phase screens was 45,683 compounds.

To analyze the interaction of the screened ZINC natural compounds based on the pharmacophore hypothesis, molecular docking was performed using Glide docking tiers against MNK-2 and PIM-2, as shown in the next section.

### 3.2 Molecular docking

Molecular docking determines the binding affinity of a molecule toward the active site of its target. First, the RMSD values of re-docking of the co-crystalized ligand references with MNK-2 (ID: **2hw7**) and PIM-2 (ID: **4x7q**) were 0.1886 and 0.2791 Å, respectively indicating the accuracy and efficiency of the docking protocol utilized in this study.

The results of the Phase screen were then processed at different stages of molecular docking with the two test targets; PIM-2 and MNK-2 using Schrodinger’s Glide tool. The compounds were filtered using three Glide docking modes, HTVS, SP, and XP (Figure 1). The three docking modes differ in speed, accuracy, and scoring function. HTVS (rate: molecule/2 s) and SP (rate: molecule/20 s) have a similar evaluation function, but HTVS allows quick screening of compounds, reducing the number of intermediate conformations, and final torsion refinement, and sampling. XP docking mode uses extensive sampling compared to SP mode, which removes false positive molecules (speed: molecule/2 min). In addition, XP penalizes molecules with reduced complementarity with the binding cavity of the receptor.

The ligands bound to the crystal structures of the two targets were considered references and were subjected to XP docking. The two references showed XP docking scores of −10.755 and −7.793 kcal/mol for the targets MNK-2 and PIM-2, respectively. These values were considered as the threshold for the selection of hits for further stages of docking. Thereafter, the results of the Phase screen for each of the five hypotheses were subjected to molecular docking against the active site of the MNK-2 (PDB ID: 2hw7). Compounds with a docking score ≤ −10.755 kcal/mol were classified as group A (62 compounds).

On the other side, the compounds obtained from the Phase screen of the five hypotheses were also docked into the active site of the second target; PIM-2 (PDB ID: 4x7q). They also passed through HTVS, SP, and XP modes of Glide. All resulting 424 compounds with docking scores ≤ −7.793 kcal/mol were classified as group B. Then, group A and group B were combined into group C (486 compounds); which included all compounds with docking scores better than the two reference ligands of the two targets in the present work; MNK-2 and PIM-2. In the second docking stage, group C compounds were re-docked against MNK-2 and PIM-2. The top 5 compounds with the best docking scores against the two targets are presented in Table 1.

**FIGURE 3:**
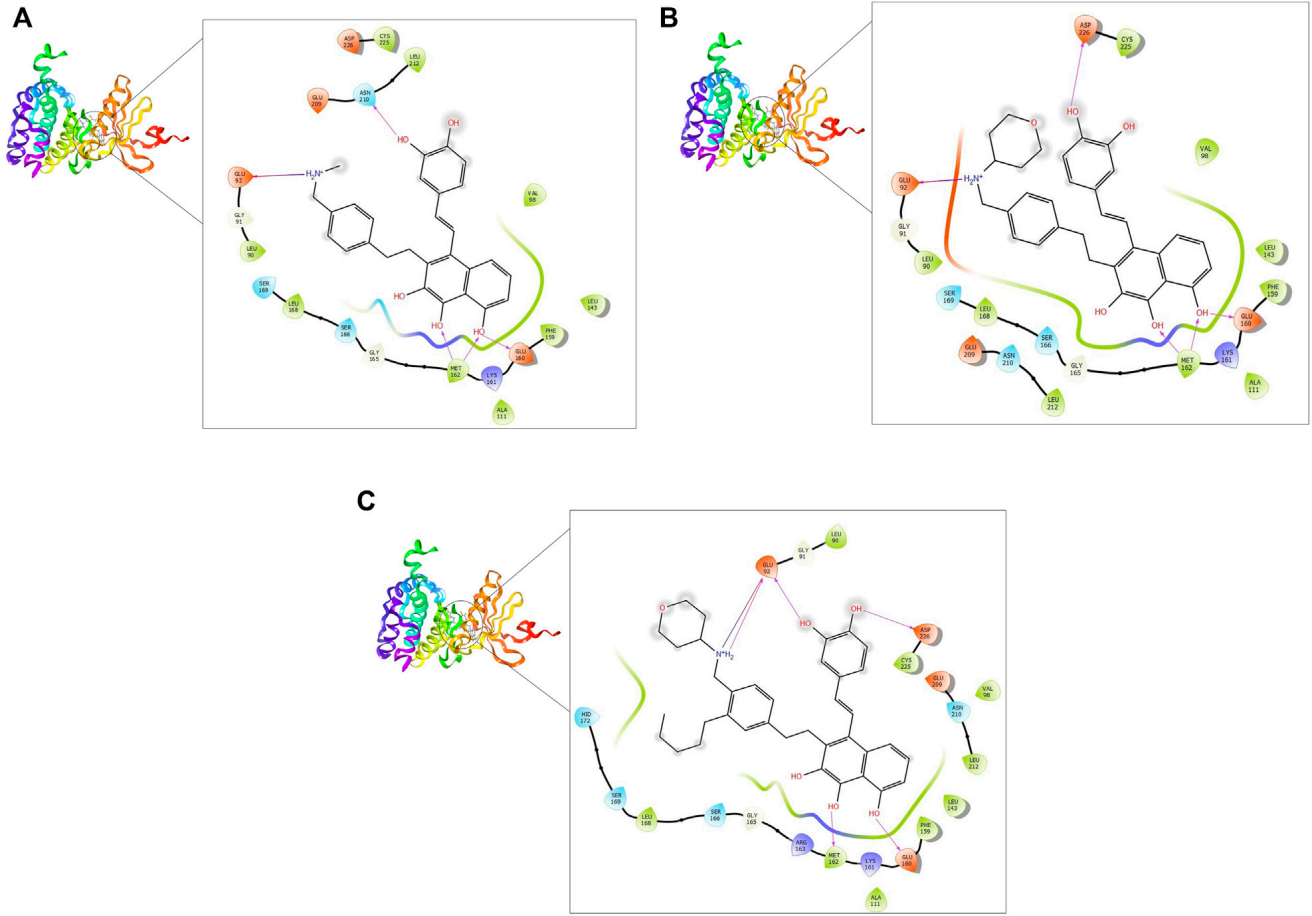
2D and 3D interaction of the top three compounds in complex with MNK-2 (ID: 2hw7) using XP docking mode of Glide software. **(A)** compound 1, **(B)** compound 2, and **(C)** compound 3. The hydrogen-bond interactions with residues are represented by a purple dashed arrow directed towards the electron donor. The hydrophobic residues are in green color.

**FIGURE 4:**
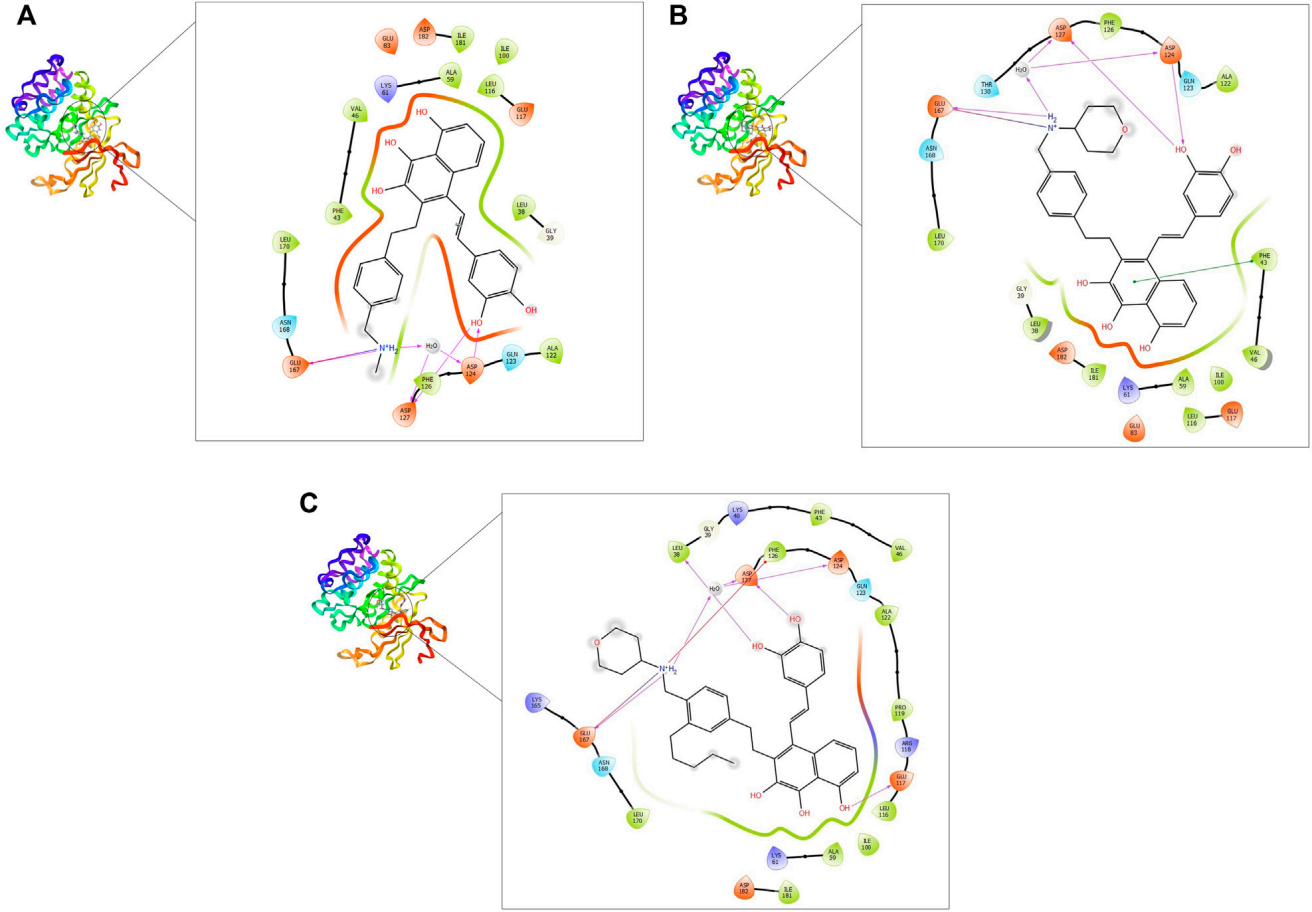
2D and 3D interaction of the top three compounds in complex with PIM-2 (ID: 4x7q) using XP docking mode of Glide software. **(A)** compound 1, **(B)** compound 2, and **(C)** compound 3. The hydrogen-bond interactions with residues are represented by a purple dashed arrow directed towards the electron donor. The hydrophobic residues are in green color.

The five compounds ZINC000085569211, ZINC000085569178, and ZINC000085569190, ZINC000008879593 and ZINC000012886855 were designated as compounds 1, 2, 3, 4 and 5, respectively in this manuscript. Compounds 1, 2, and 3 were selected for further analysis based on their docking scores and MM-GBSA binding free energy against MNK-2 and PIM-2 (Table 2 and Figures 3, 4).

**TABLE 2:** Molecular interactions of the top three compounds ZINC000085569211, ZINC000085569178, and ZINC000085569190 with MNK-2 (PDB ID: **2hw7**) and PIM-2 (PDB ID: **4x7q**).

Target	Ligand	Pi-cation interaction	Pi-pi interaction	Hydrogen bonding interaction	Hydrophobic interaction	Water bridge
MNK-2	ZINC000085569211	—	—	ASN210, GLU92, GLU160, MET162	CYS225, LEU212, VAL98, LEU143, PHE159, ALA111, MET162, LEU168, LEU90	—
	ZINC000085569178	—	—	ASN226, GLU92, GLU160, MET162	CYS225, VAL98, LEU143, PHE159, ALA111, MET162, LEU212, LEU168, LEU90	—
	ZINC000085569190	—	—	ASP226, GLU92, GLU160, MET162	LEU90, CYS225, VAL98, LEU212, LEU143, PHE159, ALA111, MET162, LEU168	—
PIM-2	ZINC000085569211	—	PHE43	ASP124, ASP127, GLU167	ALA122, PHE126, LEU170, PHE43, VAL46, ILE100, LEU116, ALA59, ILE181, LEU38	ASP124, ASP127
	ZINC000085569178	—	PHE43	ASP124, ASP127, GLU167	LEU170, HE126, ALA122, PHE43, VAL46, ILE100, ALA59, LEU116, ILE181, LEU38	—
	ZINC000085569190	PHE126	—	ASP127, LEU38, GLU167, GLU117	LEU38, PHE43, VAL46, PHE126, ALA122, PRO119, LEU116, 1LE100, ALA59, ILE181, LEU170	ASP124, ASP127

MNK-2- compound 1 complex showed five hydrogen bonds (H-bonds). Of which, two H-bonds with MET162 and three H-bonds with residues ASN210, GLU92, and GLU160. It also had nine hydrophobic interactions with amino acids CYS225, LEU212, VAL98, LEU143, PHE159, ALA111, MET162, LEU168, and LEU90 as shown in Figure 3A. Compound 2 also formed five H-bonds with the MNK-2; two of them were with amino acid MET162 and the other three with ASN226, GLU92, and GLU160. It also formed nine hydrophobic interactions similar to compound 1 with the residues CYS225, VAL98, LEU143, PHE159, ALA111, MET162, LEU212, LEU168, and LEU90 (Figure 3B). The third compound 3-MNK-2 complex revealed six H-bonds; three of them with amino acids ASN226, GLU160, and MET162, the remaining three were with amino acid GLU92, and nine hydrophobic interactions with LEU90, CYS225, VAL98, LEU212, LEU143, PHE159, ALA111, MET162, and LEU168 (Figure 3C). Here it can be seen that three ligands showed similar patterns of binding to the protein with a small difference in the number of H-bonds. These interaction patterns were also consistent with the results of several studies; H-bond with amino acid MET162 was described by K. Xing *et al.*, and X. Jin *et al.,* (Jin et al., 2019; Xing et al., 2020). Hydrophobic interactions with VAL98, ALA111, LEU168, and LEU212 were reported by S. Wang et al. (Wang et al., 2018)

On the other hand, the three ligands interacted with the PIM-2 as shown in Figure 4. Compound 1 formed four H-bonds, two with GLU167 and two with ASP124 and ASP127. Ten hydrophobic interactions with the amino acids ALA122, PHE126, LEU170, PHE43, VAL46, ILE100, LEU116, ALA59, ILE181, and LEU38, one pi-pi interaction with the PHE43, and two water bridges with ASP124 and ASP127 were also observed (Figure 4A). The second ligand, compound 2, showed four H-bonds and hydrophobic interactions similar to those of compound 1 (Figure 4B). Moreover, the compound 3-PIM-2 complex exhibited five H-bonds; two with GLU167 and the other three with ASP127, LEU38, and GLU117 as in Figure 4C. The interaction with the residue GLU117 was consistent with the result of the study carried out by Adnane *et al.*, (Aouidate1 et al., 2018). These interactions along with the good docking scores of these ligands against MNK-2 and PIM-2 gave insight that they are promising dual inhibitors.

To have a better understanding of the obtained pharmacophore hypothesis and the docking results, we mapped all five compounds (1–5) onto one representative pharmacophore hypothesis (Supplementary Figure S2, Supplementary Material). The interaction patterns of the pharmacophoric features of each ligand with the two targets were analyzed and discussed herein. The representative model consists of one hydrophobic (H), one positive ionic (P), and two aromatic rings (R) features. Based on the structural similarities, the five compounds can be classified into two groups, one group contains 1‒3 and the other contains 4 and 5. Likewise matching with the pharmacophore model features was observed within each group. To this end, compounds 1‒3 aligned with the positive ionic region *via* the secondary amino group while compounds 4 and 5 used the tertiary amino group for such an alignment. The alkene moiety in compounds 1‒3 accounts for hydrophobic alignment and since compounds 4 and 5 lacks alkene functionality they lied with the hydrophobic portion of the model with the methyl group attached to the aromatic heterocyclic ring. The aromatic feature of the model appeared to match the naphthalene ring in compounds 1-3 and the furo-chromenone core of compounds 4 and 5.

Analysis of protein-ligand complexes of compounds 1‒3 revealed that the aromatic feature of these compounds is required to bind, *via* hydrophobic interaction, ALA111, LEU143, PHE159, and MET 162 in MNK-2 and ALA59, ILE100, LEU116 and ILE181 in PIM-2. On the other hand, the heterocyclic core of compounds 4 and 5 interacted with the hydrophobic amino acids VAL98, ALA111, LEU143, and MET162 in MNK-2 and ALA59, ILE 100, and LEU 116 in PIM-2. The salt bridge and direct or bridged hydrogen bonds formed between the ammonium ion with GLU92 in MNK-2 and GLU167, ASP127, and ASP124 in PIM-2 clearly rationalizes the essence of the positive ion pharmacophoric feature of our established model. The positive ion feature of the other two compounds (Martelli et al., 2007; Gao et al., 2018) interacted *via* bridged hydrogen bonds with GLU92 in MNK-2 and GLU167, ASP163 and ASP182 in PIM-2. The hydrophobic feature is associated with the alkene moiety in 1-3 and the aromatic methyl group in 4 and 5. This feature was shown to interact with LEU90 or ILE181 in the active site of MNK-2 and PIM-2, respectively.

Although all compounds 1‒5 meet the required pharmacophoric feature for inhibiting the two targets, docking scores showed that 1‒3 had better binding affinity compared to 4 and 5. The XP Glide docking score takes into account many factors, among them the number of formed hydrogen bonds and rotating bonds (ROB) (see Section 2.4).

The top three compounds (1, 2, and 3) have 13, 14, and 18 ROBs, respectively, while compounds 4 and 5 have 6 and 7 ROBs, respectively. Furthermore, only two hydrogen bonds were observed for the interaction of compounds 4 and 5 with MNK-2 at the time compounds 1‒3 interacted with this target *via* at least five hydrogen bonds.

With respect to the interaction with PIM-2, compound 3 displayed seven direct and bridged hydrogen bonds, compound 1 formed 6 direct and bridged hydrogen bonds. Only two direct hydrogen bonds were seen for compounds 2, 4, and 5.

### 3.3 MM-GBSA binding energy calculation

In computational drug discovery methods, the binding mode of the ligand-protein complex is interpreted by docking simulation. However, the binding affinity and stability of certain complexes cannot be measured by docking only. Thus, post docking investigation must be employed to avoid any false-positive results (Azam et al., 2022). The binding affinity of the top five ligand-protein complexes was calculated to ensure the accuracy of docking screens by using post-docking free binding energy calculation (MM-GBSA) (Lin et al., 2021). MM-GBSA is considered an important technique that estimates the binding affinity where the higher negative energy values indicate greater complex affinity (Al-Karmalawy et al., 2021). The MM-GBSA for the top five compounds was calculated against both MNK-2 and PIM-2. The MM-GBSA free binding energy of the top five ligand-MNK-2 complexes was found in a range from −49.29 to −64.73 kcal/mol which showed the greatest affinity compared to that of MNK-2 co-crystallized ligand (−44.22 kcal/mol) as shown in Table 1. On the other hand, energy values for the same ligands bound with PIM-2 were found in a range from −43.23 to −47.72 kcal/mol which indicates the high binding affinity of these hits to the PIM-2 as shown in Table 1.

In terms of binding free energy with MNK-2 and PIM-2, three ligands were chosen as the best hits, namely, compound 1 (−61.55 and −47.5 kcal/mol), compound 2 (−62.65 and −47.18 kcal/mol), and compound 3 (−64.73 and −47.72 kcal/mol) and were, thus, selected for ADME prediction. Being of relatively higher affinity to MNK-2 compared to PIM-2’s, these compounds were processed for MD simulations against MNK-2 only.

### 3.4 ADME prediction

The success of the drug candidates is achieved by obtaining a finely adjusted combination of safety, biochemical behavior, high selectivity and efficacy, and a desirable ADME profile (absorption, distribution, metabolism, and excretion). An ideal drug should be appropriately taken into the body, appropriately transported into different tissues and organs, absorbed in a way that does not immediately diminish its activity, and is appropriately removed (Jana and Singh, 2019).

The ADME profile of drug-like compounds is critical in drug discovery. The QikProp module of Maestro was used to predict the ADME properties of the top three hits. Many key ADME descriptors/properties were used to assess the suitability of these hits as potential clinical candidates and the predicted pharmacokinetic features are listed in Table 3. Compounds 1, 2, and 3 were anticipated to have an acceptable lipophilicity profile (QPlogPo/w: 3.141, 3.781, and 5.148) with balanced aqueous solubility (QPlogS: −3.535, −4.845 and −5.611), respectively. Human oral absorption (HOW) was significantly low at a value of 1 for each of them, this value is in good agreement with the values of the compound’s permeation through several barrier models QPPCaco (18.883–19.986) and QPPMDCK (7.818–7.971). Moreover, the low QPlogBB value in the range between −2.047 and −2.594 reflects a limited effect on the CNS (QikProp’s Manual, 2015).

**TABLE 3:** ADME analysis of the top three hits.

Predicted ADME descriptors	ZINC000085569211	ZINC000085569178	ZINC000085569190
QPlogPo/w (−2.0 to 6.5)	3.141	3.781	5.148
QPlogS (−6.5 to 0.5)	−3.535	−4.845	−5.611
CIQPlogS (−6.5 to 0.5)	−6.078	−6.915	−8.312
QPPCaco (<25 poor >500 great)	19.986	18.883	19.63
QPlogBB (−3.0to 1.2)	−2.047	−2.353	−2.594
QPPMDCK (<25 poor >500 great)	7.971	7.497	7.818
HumanOralAbsorption (1, 2, or 3 for low, medium, or high)	1	1	1

### 3.5 MD simulations

The top three hits (compounds 1–3) were selected from the final shortlisted candidates for MD simulations studies against MNK2 protein. The protein-ligand complex’s steady nature and conformational stability were evaluated during 100 ns simulation (Śledź and Caflisch, 2018).

#### 3.5.1 Stability assessment

**FIGURE 5:**
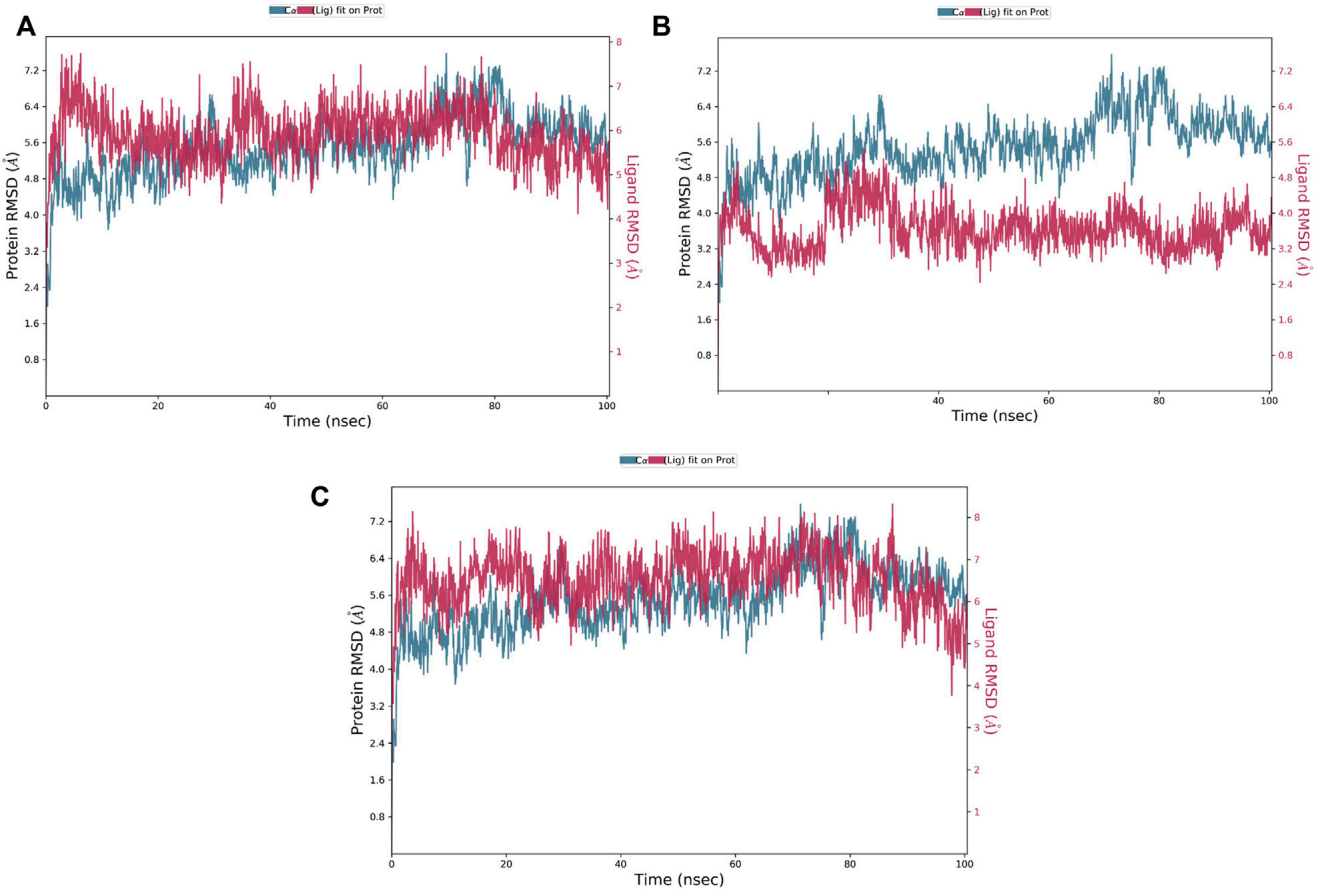
The protein-ligand RMSD plot of the top three compounds complexed with MNK-2 (ID: 2hw7) during 100 ns molecular dynamics simulation using Desmond software. **(A)** compound 1, **(B)** compound 2, and **(C)** compound 3.

The RMSD is a critical parameter for assessing the protein system’s stability. The fluctuation expansion of the Cα atom’s RMSD curve is inversely related to the stability of the system; the smaller the fluctuation, the more stable the system (Wei-Ya et al., 2019). As shown in Figure 5, compounds 1 and 3 were strongly bound to MNK-2 compared to compound 2 which was more stable than the protein. The average RMSD of compounds 1 and 3 complexed with MNK-2 was found to fluctuate around 6.4 Å.

Root mean square fluctuation (RMSF) evaluates the degree of the displacement of a specific atom, or group of atoms, relative to the crystal structure, which is averaged over the number of atoms. Atoms with a high RMSF value possess more flexibility, whereas those with a low RMSF value possess restrained movement, reducing flexibility.

**FIGURE 6:**
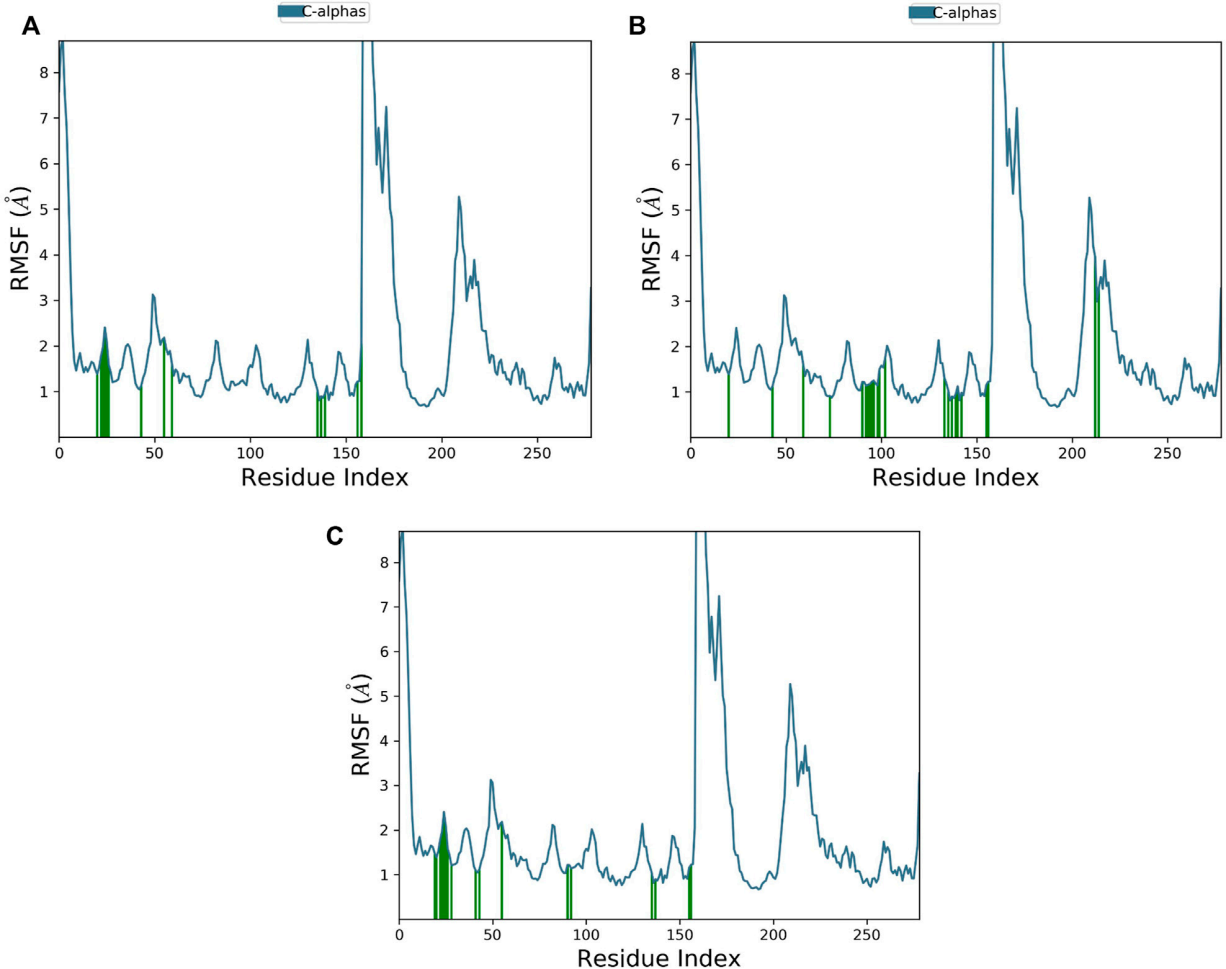
The protein RMSF plot of the top three compounds complexed with MNK-2 (ID: 2hw7) during 100 ns molecular dynamics simulation using Desmond software. **(A)** compound 1, **(B)** compound 2, and **(C)** compound 3.

The RMSF plot of Cα protein can calculate the average fluctuation of all residues during MD simulations. Small fluctuations, i.e., below 2 Ǻ, were observed for most of the interacting MNK-2 residues and high fluctuations for residues 159–175 as shown in Figure 6.

**FIGURE 7:**
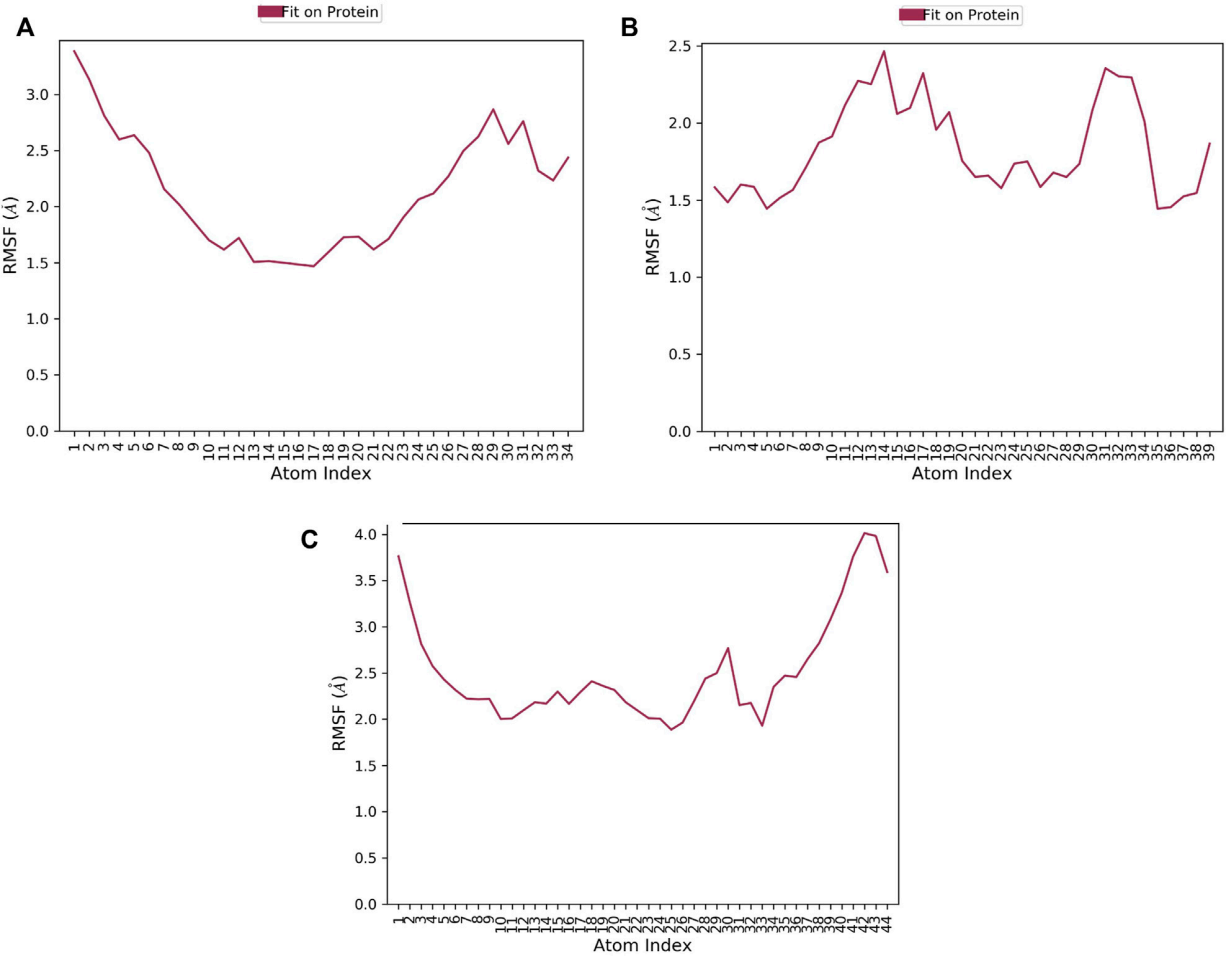
The ligand RMSF plot of the top three compounds complexed with MNK-2 (ID: 2hw7) during 100 ns molecular dynamics simulation using Desmond software. **(A)** compound 1, **(B)** compound 2, and **(C)** compound 3.

RMSF of a ligand shows the fluctuations of the ligand per atom; this could reveal how ligand fragments interact with proteins and their role in the binding process (Martínez, 2015). The average RMSF values of compounds 1, 2, and 3 were 0.36Å 0.505Å, and 0.76 Å, respectively implying the less flexibility of the three ligands with MNK-2 throughout the simulation, as presented in Figure 7. The RMSF plot of compound 1 is more stable than those of other ligands, indicating its greater stability during the simulation.

#### 3.5.2 The ligand’s interactions with the residues in MNK-2

**FIGURE 8:**
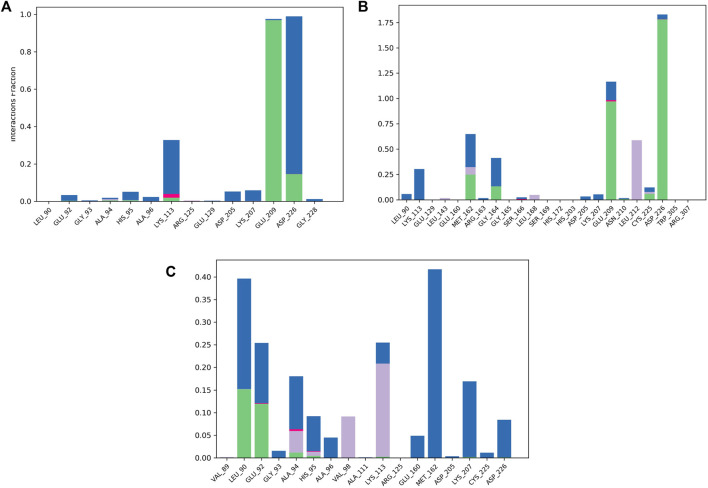
Protein-ligand contact histogram of the top three compounds complexed with MNK-2 (ID: 2hw7) during 100 ns molecular dynamics simulation using Desmond software. **(A)** compound 1, **(B)** compound 2, and **(C)** compound 3.

To validate the docking results, MD simulations were analyzed in terms of the interactions of MNK-2 and PIM-2 residues with the ligands. Figure 8 depicted the interactions in the three ligands-MNK-2 systems.

Compound 1 interacted with ASP226 (100%) (hydrogen bond (20%) and water bridge (80%)). Also, it formed a strong hydrogen bond with GLU209 (99%) and showed a water bridge interaction with LYS113 (35%). The second ligand, compound 2, displayed strong hydrogen bonding interactions with ASP226 (175%), GLU209 (100%), and water bridges with GLU209 (25%) and LYS113 (25%) revealing almost the same interactions as compound 1 with slightly different interaction strength. In addition, compound 2 also interacted with MET162 (70%) (hydrogen bond (25%), water bridge (30%) and hydrophobic interaction (15%)). In addition, it displayed hydrophobic interaction with LEU212 (55%). Compound 3 displayed water bridges with MET162 (43%), LYS207 (18%), and LEU90 (25%), hydrogen bond with LEU90 (15%), and hydrophobic interaction with LYS113 (23%).

 2, and 3 showed higher affinity to MNK-2 than Staurosporine, the co-crystallized ligand, as judged by docking scores (−12,578, −12,174, −12,113, and −10.755 kcal/mol, respectively) and MM‒GBSA values (−61.55, −62.65, −64.73 and −44.22 kcal/mol, respectively). Although these hits docked into the PIM-2 active site with scores better than that for the co-crystalized ligand, 3YR, (−10.612, −10.907, −10.022, and −7.793 respectively) binding energy calculations revealed that it has much less affinity to the target than 3YR (−47.5, −47.18, −47.72 and −70.37 kcal/mol). Nonetheless, these hits found of less affinity to PIM-2 when compared to the co-crystallized ligand (3YR). ADME processes were performed for these hits and the results showed that the predicted pharmacokinetic profile is satisfactory as all values were within acceptable limits. Being of higher affinity to MNK-2 than to PIM-2, a molecular dynamics study was used to evaluate the interaction stability and flexibility of these ligands with MNK-2. Interestingly, all ligands proved to be of good stability (measured by RMSD) and proper flexibility (measured by RMSF) with compounds 1 and 3 being the best.

## 4 Conclusion

Nowadays, *in silico* studies are considered one of the fundamental drug discovery and development methods that play an essential role in the identification and screening of new drugs. The current study explains the potential binding affinities of the ZINC natural compounds against PIM-2 and MNK-2 of AML. Starting from 270540 compounds, 45683 were found to match the hypotheses features of the co-crystallized ligands of PIM-2 and MNK-2. Furthermore, these compounds were subjected to a two-stage docking analysis that enabled us to expect the binding mode of the best hits by unveiling different types of chemical interactions with the amino acid residues at the active site of each target. Five hits were predicted to inhibit the two targets. After refining, the best 3 hits compound 1 (ZINC000085569211), compound 2 (ZINC000085569178), and compound 3 (ZINC000085569190) interacted with MET162, GLU92, and GLU160 through hydrogen bonds at the active site of MNK-2, where they formed hydrogen bonds with ASP124, ASP127, GLU167, and GLU 117 at the binding pocket of PIM-2. Docking results alone are not enough for determining the binding affinity of these compounds, therefore, MM-GBSA and MD analyses were performed not only to constraint the reliability of docking findings, but also to elucidate the affinity and stability of these compounds with the targets. Compounds 1‒3 displayed favorable MM-GBSA binding free energy and acceptable ADME properties. Compounds 1 and 3 showed stable interactions with MNK-2 during 100 ns MD simulations. Based on the study findings, these hits are suggested for future experimental investigations as novel hits for AML treatment.

## Data Availability

The original contributions presented in the study are included in the article/Supplementary Material; further inquiries can be directed to the corresponding author.

## Abbreviations

ADME, absorption, distribution, metabolism, and excretion; AML, Acute myeloid leukemia; BCRP, Breast cancer resistance protein; HTVS, high virtual screening throughput; IMP, imipenemase; MAPK, Mitogen-activated Protein Kinase; MD, Molecular dynamics; MM-GBSA, Molecular Mechanics Generalized Born and surface area; NP, Natural products; PRCG, Polak-Ribier Conjugate Gradient; RMSD, Root mean square deviation; RMSF, Rootmean square fluctuation; SP, standard precision; XP, extra precision.
