# Supplementary material for: Discovery of novel natural products as dual MNK/PIM inhibitors for acute myeloid leukemia treatment: Pharmacophore modeling, molecular docking, and molecular dynamics studies
Source: Front Chem. 2022 Jul 22;10:975191. doi: 10.3389/fchem.2022.975191 (PMC9354516; doi:10.3389/fchem.2022.975191)
Supplement: Supplementary file 1 [file DataSheet1.docx]

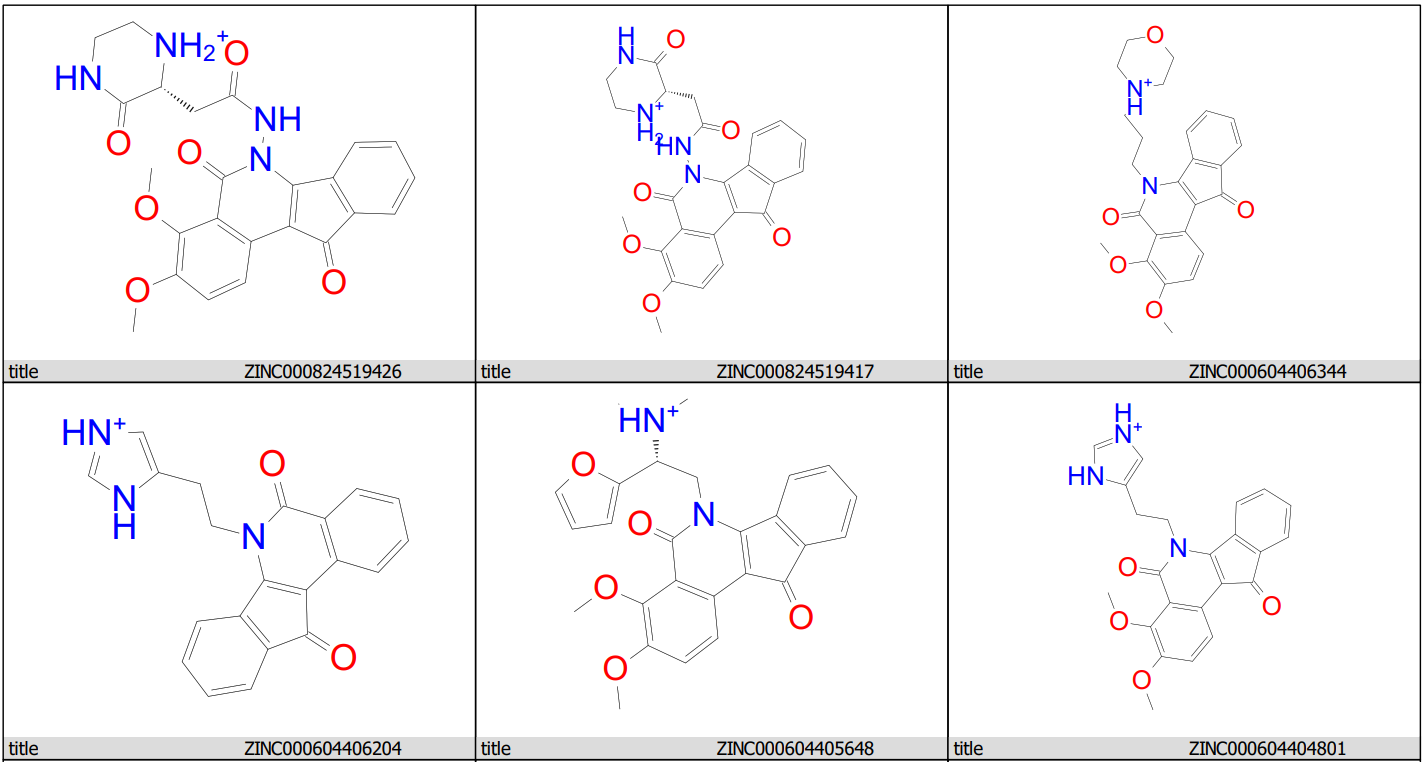


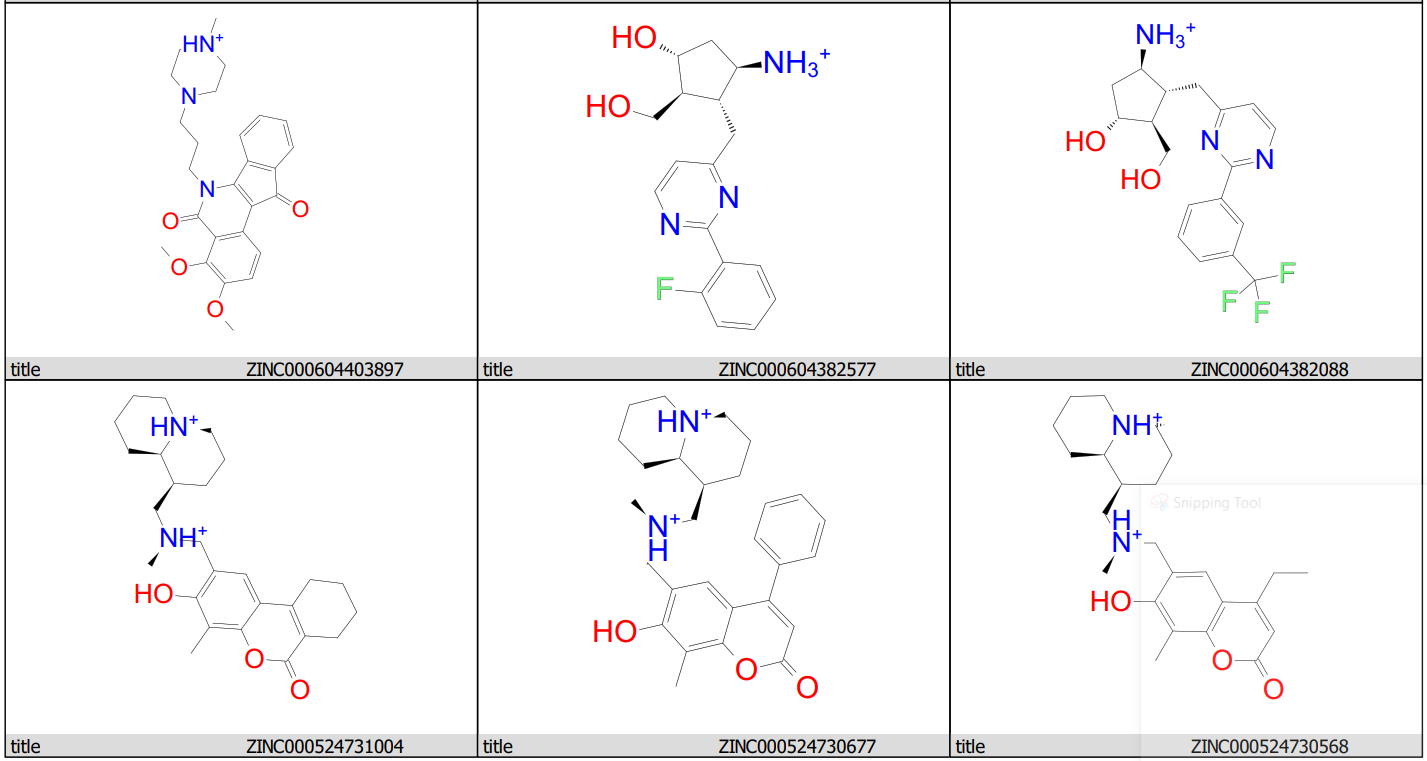


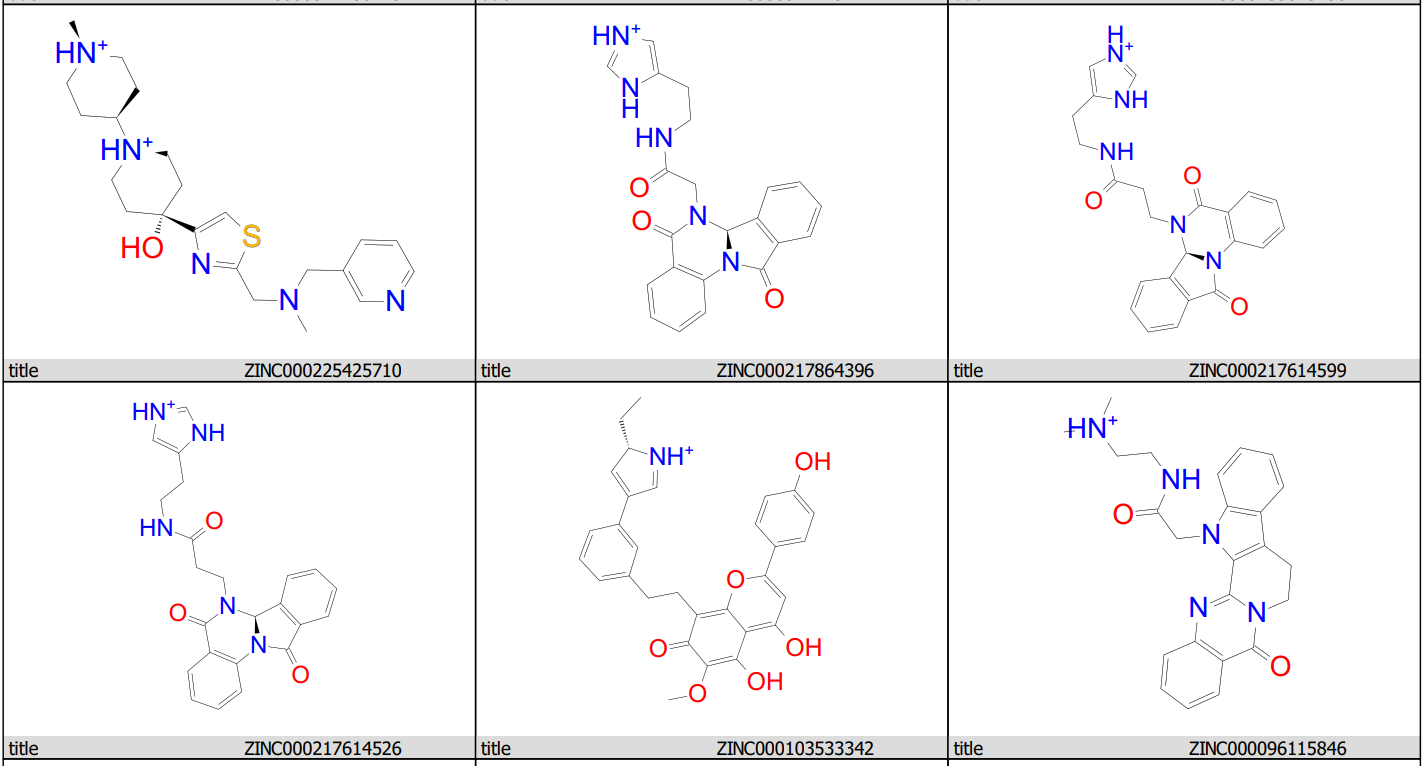


**Figure S1:** Representative natural compounds from the Zinc database that used in this study.


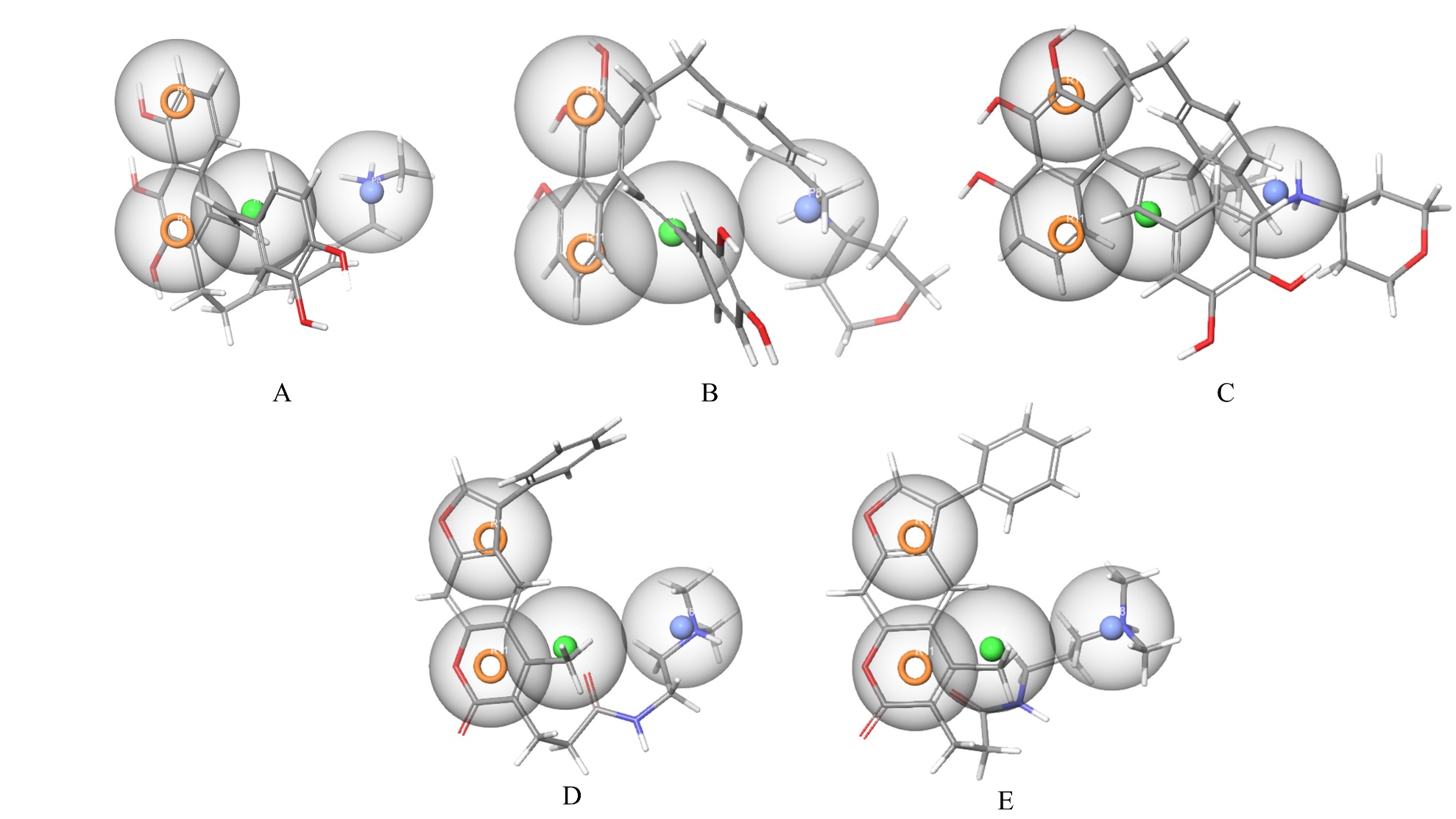


**Figure S2:** Mapping of compounds 1-5 onto the pharmacophore hypothesis. A: compound 1, B: compound 2, C: compound 3, D: compound 4, and E: compound 5. (H) hydrophobic group, green sphere; (P) positive ionizable group, violet sphere; (R) aromatic ring, yellow circle.
